# The concept of social dignity as a yardstick to delimit ethical use of robotic assistance in the care of older persons

**DOI:** 10.1007/s11019-021-10054-z

**Published:** 2021-11-25

**Authors:** Nadine Andrea Felber, Félix Pageau, Athena McLean, Tenzin Wangmo

**Affiliations:** 1 Institute of Biomedical Ethics, Faculty of Medicine, University of Basel, Basel, Basel, Switzerland; 2 Institute of Applied Ethics, Faculty of Philosophy, Faculty of Medicine, University Laval, Quebec, QC Canada; 3 Department of Sociology, Anthropology and Social Work, Central Michigan University, Mount Pleasant, MI USA

**Keywords:** Biomedical ethics, Robots, Caregiving, Elderly, Dignity, Activities of daily living (ADL)

## Abstract

With robots being introduced into caregiving, particularly for older persons, various ethical concerns are raised. Among them is the fear of replacing human caregiving. While ethical concepts like well-being, autonomy, and capabilities are often used to discuss these concerns, this paper brings forth the concept of social dignity to further develop guidelines concerning the use of robots in caregiving. By social dignity, we mean that a person’s perceived dignity changes in response to certain interactions and experiences with other persons. In this paper, we will first present the concept of social dignity, and then identify a niche where robots can be used in caregiving in an ethical manner. Specifically, we will argue that, because some activities of daily living are performed in solitude to maintain dignity, a care recipient will usually prefer robotic assistance instead of human assistance for these activities. Secondly, we will describe how other philosophical concepts, which have been commonly used to judge robotic assistance in caregiving for the elderly so far, such as well-being, autonomy, and capabilities, are less useful in determining whether robotic assistance in caregiving is ethically problematic or not. To conclude, we will argue that social dignity offers an advantage to the other concepts, as it allows to ask the most pressing questions in caregiving.

## Introduction

In an attempt to improve caregiving, both for the patient and the caregiver, robots are being introduced into the healthcare system (Vandemeulebroucke et al. 2018). So far, their tasks include gathering information about patients (Kim et al. 2013; Putte et al. 2019), assisting people with dementia through prompts in activities of daily living (ADLs; Wang et al. 2017), or to mobilize patients by interacting with them (Pfadenhauer and Dukat 2015), among others. These tasks have one feature in common: in order to execute them, the robots “socialize” with the care recipients, meaning that they interact with them in ways that mimic interactions with other persons or animals. They are thus social robots (Sparrow and Sparrow 2006). The introduction of social robots has caused worries about its ethical consequences, especially in regards to replacing human caregiving. The different concerns range from fear of isolation and loss of human contact, to questions about privacy (Pirhonen et al. 2020; Sharkey 2014; Sharkey and Sharkey 2012; Sparrow and Sparrow 2006; Wangmo et al. 2019).

Our paper adds to the ethical discussion of using robots in caregiving for the elderly in two ways. Firstly, drawing from the concept of social dignity, we suggest a specific way in which robotic assistance can mitigate the fear of replacing human caregiving, and thus be ethically less problematic. We highlight that because dignity is a social concept and depends on how we interact (or not) with others, some caregiving tasks can be outsourced to robots. The reason being that the presence of a robot could be more dignifying than the presence of a human caregiver. These tasks include (and are possibly not limited to) all areas of private personal hygiene, from bowel and bladder movements to personal cleaning. By looking at these ADLs (which we will call “non-social ADLs” in this paper) through the lens of dignity, we argue for increased efforts in developing robots that would provide helpful assistance in certain ADLs. Secondly, we will present how different philosophical concepts, such as well-being, capabilities and autonomy, are rather unreliable in offering guidance as to whether robotic assistance in caregiving is ethically problematic or not, especially in regards to the fear of replacing human caregivers. Thus, we will return to the concept of social dignity, to show that it is actually a better concept to help us judge in the matter of robotic caregiving, as it focuses more clearly on the ethical issue at hand. In the end, our arguments should convince readers that the ethical challenge of using social robots is untangled slightly thanks to the concept of social dignity, and that robotic assistance in certain ADLs might not only be ethically permissible, but in fact desirable, as it would increase the sense of dignity in both patients and caregivers.

## Structure

This paper is structured into four sections. We start with presenting the current landscape of robotic assistance in caregiving, showing how it is focused on mimicking behavior and interaction from persons or animals, making social robots the prevalent form of robotic assistance in current caregiving. This will be followed by ethical concerns raised in the use of social robots. While the field of caregiving is very wide and diverse, this paper will focus on the context of caregiving for older persons. This will be followed by the introduction of social dignity as a concept. We will elaborate on its usefulness in regards to the decision of robotic assistance in caregiving for the elderly, especially because it allows the identification of a specific area where robotic caregiving might actually be desirable. Third, we discuss the concepts currently used to discuss social robots in caregiving, such as well-being, autonomy, and capabilities, and show how they rest indecisive. This is where we want to circle back to social dignity, and propose an alternative to look at the ethical problems at hand. Finally, we offer a discussion in which we put our arguments in the current caregiving context, hoping to inspire researchers and engineers to take a new direction in the development of assistive robots in caregiving.

## The current landscape of robotic assistance in caregiving and associated ethical concerns

### Robotic assistance in caregiving

As has been mentioned in the introduction, robots^1^ used in caregiving and well-known examples include the humanoid robot, Pepper (Mubin et al. 2018), and Paro, the robotic baby seal (Pfadenhauer and Dukat 2015). Both are fashioned after living beings with whom we can intuitively interact and socially bond (albeit Paro was specifically built after an animal that is not a common pet, in order to increase acceptance, Pfadenhauer and Dukat 2015). Paro’s baby seal appearance is specifically designed to solicit the same response as if it were a puppy or another cute, care-needing animal. In addition, it reacts to sounds and touch, like a pet (Pfadenhauer and Dukat 2015). Pepper has a human-like face, where eyes, mouth and ears are located in a familiar way for humans for quick association. Pepper interacts through hearing, seeing, speech and motion with his environment (Mubin et al. 2018). The purpose of both social robots is the engagement of the user, in order to entertain them and maintain physical and mental functioning. Furthermore, Paro was designed to increase older persons’ well-being and capabilities, especially those with dementia (Pfadenhauer and Dukat 2015).^2^

Pepper and Paro are no exception, but rather the well-known standard in current robotic caregiving. A potentially caregiving robot developed in South Korea performed an (albeit fake) eye exam on patients, interacting with them through speech and movement (Kim et al. 2013). Another humanoid robot tested in Canada delivers spoken prompts to people with dementia, helping them fulfill tasks such as making tea or washing hands (Wang et al. 2017). Many more examples of social robots could be cited (Sharkey and Sharkey 2012), however, they are not yet as established as Pepper or Paro.

The fact that social robots are the main trend in robotic assistance in caregiving is further shown through studies in the field of sociology, psychology, nursing and medicine. These studies focus on the impressions and experiential impacts of such robots on patients and caregivers (Boumans et al. 2019; Law et al. 2019; Łukasik et al. 2020), and often even move a step further to imagine future uses or possibilities. For example, in a pilot study with a robot which gave prompts to older adults with dementia, older participants (as well as their informal caregivers) were asked about acceptance and future use of such a social robot, the result being that older adults were curious about the robot, but reluctant to use one in their home (Wang et al. 2017). In the study with the ophthalmology-robot, which performed the eye-check, psychosocial implications were measured, to test future use of these robots (Kim et al. 2013). Lee et al. (2018) questioned nurses about their needs and expectations in regards to robotic assistance and what they wish and fear for the future concerning robotic assistance. They found that the nurses expected help primarily in measuring/monitoring, mobility/activity, safety care, and hoped for a reduction in workload. Law et al. (2019) concluded that people with mild dementia had mixed feelings about introducing an assistive robot into their lives and homes, and that they wished for more personalization in the robot, for example in regards to the preferred activities of the patients, their personal history, or their specific medication.

Such research underlines the growing interest and incorporation of social robots in caregiving, but also reasons for caution. For example, these studies also point towards some aversion to the adoption of such robots in caregiving context. Patients are not alone in questioning the adoption of robotic caregivers (Putte et al. 2019; Wang et al. 2017). Professionals also mention the risk of harming the relationship between patient and caregiver by introducing robots, fearing the loss of an important relational element of their work (Lee et al. 2018), or by harming the patient through deception and isolation (Wangmo et al. 2019).

### Ethical concerns with social robotics

Alongside these practical concerns of caregivers and other professionals, many ethical issues are raised in the literature: Topics such as privacy (both personal and informational, as is it permissible to subject an older person to technological surveillance in order to ensure her safety?), justice (fair access to these technologies or who gets access to what kind of assistance?), responsibility (who is in charge if something goes wrong with the technology, the user or the developer, the programmer or the machine?) and affordability (how expensive will robots be? Circling back to the question of justice) have been discussed by a variety of scholars (Felzmann et al. 2015; Mittelstadt 2017; Ienca et al. 2018). While all of these concerns are valid, we will focus on one of the most often raised issues, which is the replacement of human care (Sharkey and Sharkey 2012; Borenstein and Pearson 2010; Ienca et al. 2018; Zwijsen et al. 2011).

Many scholars have been examining the trend of social robotics in caregiving in depth to find answers to this pressing question (Pirhonen et al. 2020; Sharkey 2014; Sharkey and Sharkey 2012; Sparrow and Sparrow 2006; McLean 2011). For example, Sparrow and Sparrow (2006) state “Our strong suspicion is that, regardless of the intentions of the designers and manufacturers, in reality robots will inevitably be used to replace human staff” (p. 150). And Sharkey and Sharkey (2012) point out: “[Robots] cannot form adequate replacements for human love and attention. Unfortunately, this does not mean that they will not be used as such (…)” (p. 35).

Despite these worries, scholars are nevertheless not ready to dismiss robotic caregiving altogether, as they recognize its potential. Borenstein and Pearson (2010), for example, write “In addition to relieving some of the frustration, awkwardness, and sense of dependence associated with requesting assistance from other persons, the presence of certain kinds of robots may ease depression caused by loneliness. Even if robots do not provide genuine friendship, they may mitigate feelings of isolation” (p. 282). It is interesting to note that despite the presence of deception in the relationship between the robot and the care-receiver (Wangmo et al. 2019; Sharkey and Sharkey 2011), it is not enough to dismiss the assistance of robots. This indicates that ensuring well-being outweighs the cost of deception.^3^

As we envision a future that increasingly embraces robotic devices in elder care, it is necessary to take into account the pros, cons and possible ramifications of their adoption. If all robotic assistance were rejected as unethical, on the grounds that they substitute for human assistance, valuable opportunities for humankind might go untapped. However, welcoming all kinds of robots into our lives, without further analysis, would be quite unwise as well. The challenge is to attempt to determine what is ethical (and unethical) in using robots in caregiving. And while the authors mentioned above raise important issues and discuss them with convincing arguments, they are yet to produce any clear guidelines with regards to the use of social robots in caregiving for the elderly. Despite general agreement that robots cannot and should not replace the relationships established through human caregiving (Sharkey and Sharkey 2012, p. 29; Pageau 2019), their partial adoption in cases of absent or inadequate human contact is deemed more acceptable. Nonetheless, scholars suggest that ideally, robotic assistance should be used just an add-on to human caregiving, and not a replacement of human caregivers (Sparrow and Sparrow 2006, p. 156; Pageau 2019). The eventual shift from assistance to replacement, and circumstances that lead to it are likely to vary from case to case, which is why we introduce the concept of dignity, specifically “social dignity” into the debate, as an integrative concept to help address these issues.

## Dignity as a promising concept to assess the ethics of robotic assistance

In order to integrate robotic caregiving in an ethical way and draw appropriate boundaries of use, many scholars have relied on concepts such as autonomy (Pirhonen et al. 2020; Decker 2008), well-being (Sparrow and Sparrow 2006), and the capabilities-approach (Sharkey 2014; Law et al. 2019).^4^ Sharkey and Sharkey (2012) recognize that dignity is related to well-being as ensuring dignity will have a positive psychological effect. Pirhonen et al. (2020) also consider dignity as an adequate concept to fall back onto, once autonomy is lost or limited in a care recipient due to dementia. And the capabilities approach was actually constructed to identify the “bare minimum of what respect for human dignity requires” (Nussbaum 2006, p. 70). It is therefore worth examining how dignity might offer more adequate guidance for the use of robots in caregiving.

Sharkey (2014) notes that dignity is used in different forms and has several meanings, and that there is only a basic consensus on “(…) a distinction between (a) the inviolable or universal dignity (Menschenwürde), that is an inherent property of human beings, and which does not depend on their behaviour, their beliefs, or their circumstances, and (b) other forms of dignity which can be held to varying degrees” (Sharkey 2014, p. 65). The inviolable concept of dignity, “Menschenwürde”, is what remains in a person even when she is treated in the most disrespectful manner. It’s an inherent, untouchable characteristic of human beings, that does not depend on others, whether human or robots. Therefore, we will not focus on “Menschenwürde”, except tangentially, but on the other forms of dignity, which we call “social dignity,” because they depend on relations with others. These are discussed below in more detail:

Nordenfelt (2004) distinguishes among three notions of dignity distinct from “Menschenwürde”. There is dignity of *merit*, which is related to the social status of a person. Certain professions, job titles or other achievements will earn dignity of merit. Then there is dignity of *moral stature*, which is linked to how a person behaves morally. If she acts in contradiction to her values at any given time and loses touch with her moral self, she loses dignity as well. And lastly, there is dignity of *identity*, which is closely related to a person’s representation of oneself in society, making this dignity largely dependent on how we are treated by others.

Schroeder (2010) offers a similar distinction but gives these forms of dignities different names: dignity due to status is called *aristocratic dignity*; the one in response to values is called *meritorious dignity*; and dignity of identity is what he calls *dignity of comportment*.

Bostrom (2008), on the other hand, simply distinguishes between dignity as a *quality* and dignity as a *social status*. While the latter resembles the same as Nordenfelt’s dignity of merit and Schroeder’s aristocratic dignity, dignity as a quality “may be thought of as a virtue or ideal, which can be cultivated, fostered, respected, admired, promoted, etc.” (p. 3). This suggests a mixture of the remaining two forms of dignity, discussed by the others (Nordenfelt 2004; Schroeder 2010). The important detail is that *all these variations of dignity depend on society and people’s interaction* within it. To put it simply, if there is no society to recognize your dignity, it seems that only your “Menschenwürde” will be preserved, as it is the only form of dignity which does not vary in any shape or form, and belongs to every human being. Hence, other forms of dignity discussed above are all inherently social—they depend on relationships and interactions we experience with other persons. Jacobson (2009) even goes a step further and divides this “social dignity” (a term that she uses and which we will adopt through this paper as well), into two categories: “(…) dignity-of-self and dignity-in-relation. Dignity-of-self is a quality of self-respect and self-worth that is identified with characteristics like confidence and integrity and a demeanor described as dignified. Dignity-in-relation refers to the ways in which respect and worth are conveyed through individual and collective behavior” (p. 3).

The pressing question to ask now is if social robots suffice to respect the social aspect of “social dignity”. Given its social nature, we question the conditions under which interaction with social robots might affect a person's dignity, positively or adversely. For example, if a resident of a nursing home would only receive his meals from a very friendly, engaging Pepper, but never from a professional caregiver, would that enhance the resident’s social dignity? Or would the interaction be neutral or even harmful because the resident feels isolated and dehumanized by being fed by a robot? Sharkey and Sharkey (2012) conclude that dignity in general is not a suitable concept to ethically evaluate robots in a caregiving context because it is not clear how robots would influence older peoples’ dignity.

In contrast to Sharkey and Sharkey**,** we are not trying to give an all-encompassing judgement of the influence of robots on human dignity, but rather seek to identify ways in which an elder's dignity might be better preserved through the use of robots than human companions. In other words, sometimes we indeed have to be alone in order to preserve our sense of dignity, which is the dignity-of-self, described by Jacobson (2009). Such situations include almost all aspects of personal hygiene like emptying bladder and bowels, showering, getting dressed, to mention a few. It is therefore worth assuming that in these situations, we might perceive the presence or indeed help of another human being as an infringement on our dignity (if one is not used to such human assistance, but to complete certain tasks on their own). Robotic assistance could be experienced as less intrusive, as we are using a tool, not a person, to fulfill a task we once could perform on our own, thus preserving dignity-of-self. Delegating assistance for non-social ADLs to robots instead of human caregivers may therefore actually enhance the social dignity of the care recipient.

Furthermore, introducing robots into non-social ADLs should not promote further isolation of care recipients. That is, while we argue that some personal activities such as using the bathroom could be delegated to robotic assistance and that doing so is ethically acceptable, we delimit our argument to such personal activities that humans prefer and have been socialized to be carried out in privacy. Therefore, other personal activities such as grooming one’s hair, medical examinations, cooking and/or eating (to name a few) should most likely remain in the hands of human caregivers as interactions with other human beings is an important constituent of what makes us human. In other words, our argument should not raise the question whether social interactions with robots would replace said interactions with human beings as robotic assistance is delegated to non-social ADLs only, thus preserving dignity-of-self, and not touching on dignity-in-relation.

In addition, it is likely that not only care recipients would benefit from robotic assistance for such non-social ADLs, but that caregivers would as well. From a practical point of view, if robots could take over tasks such as those related to the bathroom, caregivers would have more time for other tasks, and ideally more time for quality interactions with the elders. Indeed, the nurses interviewed in Japan named reduction of workload and possibility of delegation their biggest hopes in regards to robotic assistance in their jobs (Lee et al. 2018). These statements are in line with other studies, which find that nurses often suffer from stress-related symptoms and burnout (Harrad and Sulla 2018; Ziegler et al. 2016), which is only worsened by their frustration at not being able to provide quality care for elder recipients.

Thus it might also be the case that caregivers would see their own social dignity preserved, thanks to the robotic assistance in non-social ADLs. This is because preservation of social dignity might result in a more receptive attitude towards situations in which a human caregiver cares for them, creating a more beneficial relationship for both parties. In addition, if personal hygiene tasks are delegated to robots, the reduction in intimate exposures might also reduce cases of sexual harassment of caregivers by care recipients, an all too common problem in caregiving situations (Kahsay et al. 2020). Further, increased emotional exhaustion of nurses coincides with increased incidents of elder abuse (Yon et al. 2019). Improving working conditions of caregivers through the use of robots may decrease abuse by professional caregivers as well, be it in nursing homes or private homes.

Lastly, a changed profile of the caregiving profession, which involves more face to face interactions, more reliance on interpersonal and social skills and medical knowledge than hygiene related cleaning skills, as well as fewer possibilities of harassment, might make the profession of a caregiver more attractive to people in general (Blomberg et al. 2013; Hebditch et al. 2020), and aid towards decreasing the shortage of professional caregiving staff (Ackerson and Stiles 2018; Fleming et al. 2003). In addition, if caregiving becomes less physically taxing, professionals might stay longer in the workforce.

The niche of non-social ADLs to preserve their dignity-of-self, is ideal to implement robotic assistance. This solves at least part of the question whether robotic assistance in caregiving of older persons is ethical, as it identifies a place where it is. Nevertheless, we do not have a clear answer yet when it comes to other caregiving tasks and robotic assistance. In the following, we will examine how other concepts are used to deal with robotic assistance in caregiving for the elderly, as they relate to social dignity.

## Limitations of common philosophical concepts for evaluating robotic assistance

The concepts of well-being, capabilities, and autonomy have been used in helping to help determine whether robotic assistance in caregiving is or is not ethical. While each concept has strengths, it also has limitations in dealing with this issue, when compared to the concept of social dignity.

### Well-being

Sparrow and Sparrow (2006) evoke the intuitive concept of well-being (without defining it) to argue that many forms of robotic care would be actually detrimental to the care recipients. They base this on the assumption that since relationships formed with robots are unauthentic and possibly even deceptive, they cannot contribute to a person’s well-being. Borenstein and Pearson (2010), on the contrary hold that robots can mitigate feelings of isolation, thus increasing well-being. These two varying arguments based in the same principle (the increase of well-being) show how ambivalent the concept can be. This is firstly due to a lack of consensus in the definition of well-being (King et al. 2014). Secondly, well-being can be subcategorized in objective and subjective, among others (Gasper 2005) which in turn complicates the discussion. For example, it is not clear in Sparrow and Sparrow’s argument if their straightforward rejection of social robots is based on the subjective experience of well-being or objective criteria. Does the substitution of human relationships with robotic companions make one objectively unwell? What if the robots treat the patient much nicer, more thoughtfully and attentively than the human caregivers? Or is the subjective well-being diminished, but only in those patients who are actually aware of the replacement, and on top of that, dislike it? This suggests that the discussion be taken on a case by case basis, in which patients have to decide for themselves if a robot increases or decreases their subjective sense of well-being.

We suggest that the above arguments evoking well-being might be better conceptualized in terms of social dignity than well-being. Issues, such as unauthenticity and possible deception, as discussed by Sparrow and Sparrow, seem more relevant to social dignity than to well-being. For instance, in the case of spousal betrayal and deception in marriage, the well-being rests unaffected as long as the betrayal is not discovered. However, dignity might be affected even before the infidelity has been discovered because the person is likely to feel humiliated retrospectively: the humiliation doesn’t only affect the moment when truth is known, but is extended to all those moments where the relationship was deemed to be authentic. In the case of Borenstein and Pearson, their argument that robots can mitigate feelings of isolation, and thus preserve well-being, can even more deeply extend to the preservation of social dignity, we would argue. This is especially true if it is non-voluntary, and also the reason why solitary confinement can be considered as will-breaking torture (Lauritzen 2012).^5^

To illustrate how social dignity avoids the ambiguity of well-being, let us look at the following example: Certain drugs induce a very pleasant state. And in a very old, frail person, one could argue that life-shortening drug-abuse is negligible, because the person has arrived at the end of her life anyway, and the pain experienced without the injection of drugs decreases the well-being of that person significantly. Thus, from a stance of well-being, it might be beneficial to offer the older people substances such as MDMA or heroin in order to keep them in a perpetually pleasant state. Their physical (they are not in pain), mental (they feel bliss thanks to the drug), and perhaps social (as they could take the drugs together with other care recipients and thus experience a collective, drug-induced high) well-being is maintained, through the constant injection of drugs. Although these last few months of an old person’s life might be pleasant, they are probably unethical, making well-being alone not suitable to decide on such a measure. Further, let us assume that it were theoretically possible that robots used in nursing homes could ensure that elders were well fed, groomed and socially engaged, and that the elders experience a sense of well-being even in the absence of any human contact. The absence of this human component, as argued by many scholars would give us pause that this arrangement, based on well-being alone, was ethically acceptable. A likely objection to such treatment is precisely that it would be undignified.^6^ Barilan’s discussion on dignity and deception raises this objection: he mentions Nozick’s famous “experience machine” and argues that it would be undignified to hook persons suffering from dementia to it, as the harm their dignity suffers through deception is greater than the benefits of endless pleasurable experiences (Barilan 2012, p. 354). Thus, invoking social dignity instead of well-being gives clearer guidance on the use of social robots in caregiving for the elderly.

### The capabilities approach

The next concept, which is the capabilities approach (Nussbaum and Sen 1993), calls for distributional justice to ensure that every person has the same capabilities to lead a flourishing life. In the caregiving context, as argued by Borenstein and Pearson (2010), this would mean maintaining previously existing capabilities (if they want to be maintained). Caregiving robots, then, could be a way to maintain such capacities: “To the extent that robot caregivers can mitigate further declines in an individual’s health, they will also help ensure that individuals have the choice to actualize capabilities rather than live as relative prisoners of their impairments or the needs or whims of human caregivers” (Borenstein and Pearson 2010, p. 281). This goes in line with our argument about social dignity. If the capacities to take care of oneself, especially in regards to tasks that are done in privacy are maintained through the help of robots, then dignity-of-self is maintained as well.

Nevertheless, approaching the issue of robotic assistance through capabilities will not give adequate guidance for robotic assistance in the social realm, for two reasons. First, the ten items on Nussbaum’s list of capabilities that need to be ensured in order to live a human life contains the following item: “Emotions. Being able to have attachments to things and people outside ourselves (…)” (2000, p. 79). This admits an attachment to robots. However, item 7 might offer grounds to reject social robots, both in its version A and B: “Affiliation. A. Being able to live with and toward others, to recognize and show concern for other human beings, to engage in various forms of social interaction (…); B. Having the social bases of self-respect and non-humiliation (…)” (ibidem, p. 79). Firstly, it all depends on whether we exclude robots from “various forms of social interaction”, and/or if we consider interactions with robots as humiliating or not, especially if they are all an (older) person is expected to get in terms of care.^7^ Second, Nussbaum does not give further guidance here, as she insists on the approach’s “*multiple realizability*” (ibidem, p. 76), meaning that the list can be adapted—to a certain extent—to a given context. This flexibility of the theory gives leeway to accommodate a variety of social standards and practices. Taking the aforementioned example, we would need to decide in advance if interactions with robots form part of the capability to build and/or maintain healthy relationships. If it is a yes, then robotic caregiving is acceptable. If it is a no, social robots should not be used in the caregiving context. Borenstein and Pearson (2012) seem inclined to a yes (p. 282). To the extent that we would substitute a robot for a human in embracing the capabilities approach, however, we are ethically challenged by the shift from robotic assistance to full replacement—something most writers found ethically unacceptable.

Let us look at an example to make the issue with capabilities clearer: Imagine that Anna visits her grandmother once a week. Anna later buys her a personal “Pepper” to interact with (and possibly monitor her). Anna views the robot as an assistance for her grandmother that engages and maintains her grandmother’s social capacities. However, if Anna stops visiting her grandmother after introducing Pepper, it’s no longer an assistance but replacement of the interactions that she would have provided. In between this continuum of actual assistance and total replacement, the issue is less clear-cut. The robot might indeed be a good excuse to cancel some of Anna’s visits, as she feels less guilty because her grandmother has a “companionship”. Nevertheless, the robot helps the grandmother’s cognitive abilities, making conversation with her more stimulating. In addition, the robot provides great conversation-material. The visits might indeed become less frequent but of higher quality, because both the grandmother and Anna enjoy them more. How should we now classify the net impact on the grandmother’s capabilities of “Emotion” and “Affiliation”? She might develop an emotional attachment to the robot, which in and of itself can be seen as positive under the capabilities approach (she cares and is emotionally engaged). However, despite liking the robot, enjoying its company, and using it as a subject for conversation with her daughter, she might feel increased disappointment because she notes Anna’s decreasing visits, and feels less affiliated to her. It is difficult to judge if the robot has been good or bad for the grandmother’s capabilities overall. Thus, the capabilities approach is rather unsatisfying in solving the problem of robotic assistance in human caregiving.

Here again, we suggest a turn towards social dignity in order to evaluate more clearly if social robots are suitable for caregiving or not. From a psychological perspective, it seems that humans quite easily create social bond with robots, even if they are not humanoid (Sung et al. 2007). The fact that the people in this study seemed unbothered by this relationship with their cleaning robot might indicate that they indeed perceive these relationships as dignified, or at least neutral. In addition, a certain emotional attachment to things is permissible in our culture. If someone has a nickname for her smartphone, or talks to her computer, we do not judge the person as having an undignified relationship with said phone or PC. As long as the relationships to non-humans do not lead to a complete rejection or termination of relationships to humans, it seems possible that robots actually preserve our dignity-in-relation, as they provide relationships which feel respectful and pleasant.^8^ Returning to the example of Anna and her grandmother, the fact that Pepper is helping to maintain the relationship between the two is considered beneficial for the grandmother’s dignity, and therefore ethically permissible.

### Autonomy

The last concept we want to discuss is autonomy. For illustration, let us use a (partial) definition by Decker (2008): “(…) the ability of persons to spontaneously adopt attitudes and carry out actions, which are in principle not predictable (cf. Mackay 1967). Personal autonomy takes place in the form of actions in the sphere of reasons. These do not have to be determined morally or, in a narrower sense, rationally. Life plans in the sense of wishes and interests constitute a typical case of personal autonomy” (p. 320).^9^ Again, the concept of autonomy can be used both to caution against caregiving robots or to encourage their use. For example, Decker himself, with reference to Kant’s categorical imperative, states that care recipients must not be treated as objects in a caregiving context, and admits the possibility that robots could potentially do that (2008, p. 323). If the robot would only provide assistance when required and desired by the care recipient, which would translate into more freedom and independence, overall autonomy could be increased through a caregiving robot (ibidem). Sharkey and Sharkey (2012) also recognize the possibility of increased autonomy for the elderly through the use of robots. Nevertheless, they foresee complications, like those associated with paternalistic programming of robotic assistance (for example, refusing to execute other tasks until the patient has taken her medicine).

The great merit of autonomy with regards to robotic assistance is that it acknowledges that care recipients are not and should not be passive in the caregiving process, emphasizing the assistive function of robots. That is, the capacity to take decisions and actions should not be undermined, and that caregiving robots need to respect, ensure, and possibly enhance this capacity. Nevertheless, using autonomy to limit the use of robotic assistance is not sufficient because autonomy (unlike the other concepts discussed in this paper) can actually be non-existent (or more precisely denied) in certain care recipients, especially in those who need more intense caregiving. Thus, respecting the autonomy of patients, one of the most upheld and unquestioned values in current bioethics,^10^ as currently conceptualized as moral autonomy in traditional bioethics, is a value that cannot be applied to all older persons. For example, people suffering from dementia are considered less autonomous, even to the point of no longer having autonomy at all, compared with healthy older people because the formers’ cognitive capabilities are limited (Pirhonen et al. 2020), a necessary condition for retaining moral autonomy. They cannot, or in some cases, are presumed to be unable to spontaneously adopt attitudes and carry out actions that are their own, as the disease clouds their judgement and thinking, even if their wishes remain and their will is intact; as long as they are unable to communicate their wishes, or are perceived as lacking good judgment, they are not honored as having autonomy. In addition, (verbal and non-verbal) communication is also often impacted. Thus, they cannot express in an autonomous fashion if they would accept a robotic caregiver or not.^11^ Furthermore, their executional autonomy might be limited as well, as introducing the robotic assistance requires some form of learning and adapting to a new situation, which could prove impossible for dementia patients. Nevertheless, some might be capable of adopting robots as their caregivers, and actually welcome it, as it would provide them with more functional autonomy^12^ indeed (Pageau 2019). For example, a robot that is serving dinner, then patiently waiting to retrieve the plate, provides more autonomy for an easily distracted nursing home resident who takes time to finish a meal, than a nurse who will hurry the resident because she has to work on a tight schedule. However, here again we necessarily wander into hypothetical fields that will be decided case by case. Some people will take the autonomous decision to adopt robots in caregiving, and have the executional autonomy to handle them. Some people will refuse to do so. And others might not even be capable of giving their consent, and neither of using the robot, as they are deprived of autonomy, due to illness, or more precisely, the limiting philosophical lens that deprives them of autonomy on assumptions that their gestures, statements or actions, are the product of pathology rather than authentic efforts to communicate their wishes (McLean 2007). In conclusion, autonomy only offers limited guidelines in the use of robots, especially in regards to populations who only possess partial autonomy or none at all.

The concept of social dignity is not so limiting. A person with dementia possesses the same dignity as another adult without the disease, and thus both must and can be treated equally when it comes to the question of whether robotic assistance is useful or not. Therefore, coming back to the previous example, we can ask ourselves if a robot fetching dinner for a person with dementia increases their social dignity (through increased functional autonomy, or more precisely, where autonomy would be denied them, by respecting and satisfying their wishes), compared to a nurse who dictates feeding time and duration for a person. We would argue that it does.

In short, the concept of social dignity is an insightful addition to the currently used concepts to discuss robotic assistance in caregiving for the elderly. Firstly, we have shown that robots can inherently preserve the dignity-of-self, which is part of social dignity, as they allow care recipients to execute a specific type of ADLs in private—that is, the non-social ADLs—without the dignity-infringing presence of a human caregiver. In addition, robotic assistance in other types of ADLs can also be judged through the lens of social dignity, as one can ask if interactions with social robots enhances or damages one’s perception of his or her social dignity. Although the concept of social dignity does not give a final verdict pertaining to ethics for all robotic assistance in caregiving, at least it boils the question down to one concept, which facilitates philosophical discussion in the future. Finally, the ethical utility of the concept of social dignity in considering issues of robotic care, can serve to highlight the serious limitations of the construct of moral autonomy as unjustly denying recognition to persons with dementia.

## Discussion

The issues addressed in this paper are relevant to the systemic issues that the caregiving sector for older adults is generally suffering from. The institutionalization, mechanization and monetarization of elder care has led to a capitalistic system where economic considerations come first, which seldom benefits the care recipient (Leidl and Stratmann 1998), and which overburdens the professional caregivers as well. Robots can alleviate the symptoms of this systematic failure, as has been mentioned above. Ideally, they also offer the chance to restructure the system, as they would liberate a great part of human potential. Nevertheless, the current situation is capable of abusing the introduction of robots, so we must remain vigilant to their adoption.

For example, if human care received by an elderly patient is limited to the non-social ADLs (i.e. personal hygiene such as bowel and bladder movements and dressing), then delegating this assistance to robots would further isolate the patient as he or she is not in need of other support. But this might result in the care recipient only interacting with caregivers during mealtimes, and even then, as has been suggested above, caregivers may continue to be rushed and stressed, thus not providing any significant interaction. This worry is mentioned by Sparrow and Sparrow (2010), who argue that the introduction of robots into caregiving will result in more patients per nurse and robot, and hence not produce the desired outcome of more quality time to spend with older patients, reducing isolation and increasing their social dignity.

In addition, even if robots would give caregiving staff more time which could then be invested into other interactions with care recipients (such as taking more time during meals, mobilization or leisure activities), this would not necessarily mean that these additional interactions and relationships formed would be indeed beneficial for the care recipients’ social dignity, as not every interaction is per definition a “good” interaction. Unfortunately, mistreatment and/or abuse of care recipients is not uncommon (Lindbloom et al. 2007; Wangmo et al. 2017) and such disrespectful treatments stand to harm the social dignity of care recipients. It is clear that there is no risk-free situation, and that vigilance is necessary from the part of the organization as well as care-organizers, be it family members or team leaders in nursing homes or organizers of caregiving services for older people still living at home, to ensure that the balance between care shifted to assistive robots and professional caregivers indeed turn out to be positive for the older care recipient.

Nonetheless is it worth noting that the technology required for such autonomous assistance in non-social ADLs does not exist yet. In addition, even if robotics would advance this far and finally offer the possibility to, for example, lift a frail elderly person from the bed, help her getting on the toilet, clean herself and then putting her safely back to bed, it is likely that the use of such assistance will require some capabilities and skills on the part of the user. The most important one will probably be cognitive ability, which is precisely the thing many elderly care recipients lose over time, unfortunately. Thus, the challenge is to also figure out how to ensure that people suffering from dementia or other illnesses will profit from such technology as well, and not pose a threat to the dignity of the patient, as in such cases he/she may likely feel treated like an object (Sharkey and Sharkey 2012). In order to prevent this from happening, users should always have the possibility to start or stop the process of receiving assistance from a robot, be it through phone applications, buttons or voice commands. Yet as mentioned before, this will require further technological advancements, as well as special skills from patients receiving such caregiving.

With regard to the issue of privacy, technological design might play a significant role in regards to perceived privacy by the users of a robot: the more human-like the machine, the more people feel observed (Lin et al. 2011). This suggests a non-humanoid design for assistive robots for non-social ADLs, in order to ensure that the users feel less disturbed by the robot. Nevertheless, just because care recipients would not feel violated in their privacy by using such a robot, does not necessarily mean that their privacy remains intact. Indeed, existing robots are already capable of remote identification and eavesdropping, just to name a few issues (Denning et al. 2009). Also, they might be vulnerable to data theft. We therefore highly recommend that robots are constructed as securely as possible, such that unwanted infringements of privacy are kept to a minimum. In addition, the presence of a human caregiver might infringe on the privacy of a care recipient as well (Roe et al. 2001). Caregivers might feel embarrassment too when they have to assist in non-social ADL’s (Wong 2005). The infringement of privacy felt by the care recipient and the embarrassment felt by the caregiver could be mitigated through the use of a non-humanoid, assistive robot, which we consider a benefit towards the perceived privacy of the care recipients. In such situations, the shame avoided through the presence of a robot, and not a human, enhances the dignity-of-self of the care recipient.

## Conclusion

In this paper, we discussed how the introduction of robotic assistance in caregiving for older persons raises ethical concerns. We then proposed the concept of social dignity as an ethical lens for considering these issues, and identified a niche where robotic assistance is ethically preferable to human assistance. This niche is located in non-social ADLs, where privacy is desired and socially expected, such as during activities related to personal hygiene. We then described how other scholars have addressed the ethical issues surrounding robotic assistance in caregiving, utilizing the concepts of well-being, capabilities and autonomy, and identified some of their limitations. We then turned to the concept of social dignity in an attempt to gain further ethical insight into the use of robotic assistance in caregiving for the elderly. We conclude that social dignity may help facilitate ethical considerations in the use of robots in elder care with regards to particular types of ADLs. By focusing on the unique circumstances and experience of each person, social dignity provides a valuable lens for considering the relevant ethical uses, thus facilitating the philosophical discussion. Our analysis may encourage designers and engineers, in conjunction with potential users, to venture further into the construction of specific social robots which assist in non-social ADLs, as this would provide notable benefits for both care recipients and caregivers, and give scholars further input in discussing the other areas in which robotic assistance can be used in the future of caregiving for the elderly.

## Data Availability

Not applicable.

## Footnotes

1 “Robots are sensumotoric machines to extend the capabilities of human action. They consist of mechatronic components, sensors and computer-based control and guidance functions. The complexity of a robot can be clearly distinguished from other machines in terms of the higher number of degrees of freedom and the variety and scope of its forms of behavior” (Christaller et al. 2001, p. 5).

2 Paro is described as a “mental commitment robot”, offering patients with dementia pleasant and mind-stimulating interactions in a non-threatening way and with minimal risk (Pfadenhauer and Dukat 2015 p. 396).

3 This stance was taken by various interviewed healthcare professionals (Wangmo et al. 2019). Yet we can ask ourselves who encounters the cost or damage of deception (the care recipient who is deceived or actually the caregiver who deceives, as he will probably feel guilty?). Further, we question whether relationships with human caregivers, although being “real”, actually have more value than a “fake” relationship with a robot per se, given that some of these real relationships involve harmful, disrespecting behavior (Sharkey and Sharkey 2012). This means that the overall renunciation of social robots in caregiving does not necessarily entail the preservation of worthy relationships for care recipients. Thus, the question becomes one of weighing potential benefits for the care recipient against potential risks for them in regards to interactions with a social, and potentially deceptive, robot. Yet this does not solve the overarching question about the ethics of using such robots, which is why we introduce the notion of social dignity.

4 This is the reason we chose to focus on these philosophical concepts, instead of the possibly more widely known four principles of biomedical ethics, which are respect of autonomy, non-maleficence, beneficence, and justice (Beauchamp and Childress 1979).

5 A current and also very concerning case in relation to isolation is the assisted suicide of the 90-year-old Nancy Russell in Toronto, who did not want to face another COVID-19-lockdown in her retirement home (Favaro 2020).

6 Various justifications in regards to dignity can be given here. For example, we can point to the “Menschenwürde” of these patients, and state that this inherent quality of human beings commands us to care for them in a humane way. But we could also mention social dignity, because if this dignity depends on the interactions we have with other human beings, depriving the elderly entirely of such interactions is harming their social dignity.

7 If the interaction with a robot is preferred to an interaction with a human caregiver, then one could argue that there is no humiliation for the older person involved, as she chose the robotic caregiver. However, if there is no choice, feelings of humiliation may be likely to arise because the older person feels abandoned by her fellow humans and “pushed off” to a robotic caregiver.

8 One could obviously argue that the pleasure of interactions with robots is not what makes them valuable for our dignity-in-relation, precisely because they cannot convey a sense of respect or worth towards humans. They are not able to “recognize” us or our value as another human being would. However, if this argument is made, we would counter with the question of relationships with animals. A dog is also unable to recognize human worth or convey respect, like a human being could. Nevertheless, such relationships can prove very meaningful to people, and tend to be perceived as dignified.

9 Note that this definition encapsulates both decisional and executional autonomy, where decisional autonomy refers to the capacity to take a decision in regards to one’s desires or wishes, and executional autonomy refers to the capacity to act upon it (Laceulle 2018).

10 An illustrative quote by Holm (2001): “If she has made an autonomous choice, and if she has adequate information about the consequences, then we should not interfere. The reasons given for this moral claim range from the Kantian to the consequentialist side of the ethical spectrum, but it is accepted by most standard accounts of biomedical ethics and health law” (p. 154).

11 Although they might still be able to express at least aversion through physical resistance or facial expressions (and thus serve as cues to establish non-consent), they are insufficient as to establish consent.

12 This type of autonomy is well-known in geriatric care and defined by the ability to take care of oneself in the activities of daily living (feeding, toileting, dressing, bathing, continence) and instrumental activities of daily living (using the telephone, shopping, preparing food, housekeeping, doing laundry, using transportation, handling medication, handling finances).
